# A hybrid 1DCNN-GRU deep learning framework for classifying caprine granulosa cell fertility potential using single-cell transcriptomics

**DOI:** 10.14202/vetworld.2025.1922-1935

**Published:** 2025-07-17

**Authors:** Thanida Sananmuang, Denis Puthier, Kaj Chokeshaiusaha

**Affiliations:** 1 Department of Veterinary Science, Faculty of Veterinary Medicine, Rajamangala University of Technology Tawan-OK, Chonburi, Thailand; 2 Aix-Marseille University, INSERM UMR 1090, TAGC, Marseille, France

**Keywords:** 1DCNN-GRU model, deep learning, differential gene expression, goat fertility, granulosa cells, single-cell RNA sequencing

## Abstract

**Background and Aim:**

Granulosa cells (GCs) are crucial mediators of follicular development and oocyte competence in goats, with their gene expression profiles serving as potential biomarkers of fertility. However, the lack of a standardized, quantifiable method to assess GC quality using transcriptomic data has limited the translation of such findings into reproductive applications. This study aimed to develop a hybrid deep learning model integrating one-dimensional convolutional neural networks (1DCNNs) and gated recurrent units (GRUs) to classify GCs as fertility-supporting (FS) or non-fertility-supporting (NFS) using single-cell RNA sequencing (scRNA-seq) data.

**Materials and Methods:**

We analyzed publicly available scRNA-seq datasets from monotocous and polytocous goats. A set of 44 differentially expressed genes (DEGs) (False discovery rate ≤0.01, log2 fold change ≥1.5) was identified and used to distinguish FS-GCs and NFS-GCs through Leiden clustering. The expression profiles of these DEGs served as input to train a hybrid 1DCNN-GRU classifier. Model performance was evaluated using accuracy, precision, recall, and F1 score.

**Results:**

The optimized hybrid model achieved high classification performance (accuracy = 98.89%, precision = 100%, recall = 97.83%, and F1 score = 98.84%). When applied to scRNA-seq datasets, it identified a significantly higher proportion of FS-GCs in the polytocous sample (87%) compared to the monotocous sample (10.17%). DEG overlap across samples further confirmed the model’s biological consistency and generalizability.

**Conclusion:**

This study presents the first application of deep learning-based classification of goat GCs using scRNA-seq data. The hybrid 1DCNN-GRU model offers a robust and quantifiable method for evaluating GC fertility, holding promise for improving reproductive selection in livestock breeding programs. Future validation in larger datasets and across species could establish this model as a scalable molecular tool for precision livestock management.

## INTRODUCTION

Fertility evaluation in goats plays a vital role in breeding programs and enhancing livestock productivity, facilitating the selection of animals with high fecundity and superior reproductive traits, such as shorter kidding intervals and larger litter sizes. Furthermore, a deeper understanding of the genetic factors influencing fertility can enhance breeding management strategies and optimize goat production systems [1]. Among several biological indicators, granulosa cells (GCs) have gained prominence as key predictors in fertility assessments. GCs are essential for follicular development and oocyte maturation, offering structural support to ovarian follicles and regulating hormonal signaling throughout folliculogenesis [2, 3]. Through a bidirectional communication system, oocytes release signals that stimulate GC proliferation and differentiation, whereas GCs provide critical nutrients and growth factors to the oocytes. Therefore, GC quality is considered a reliable indicator of oocyte developmental competence in various livestock species, including goats. Although several traditional methods are available to evaluate GC quality, they often fail to reliably predict oocyte competence in goats [4–7].

Ligand–receptor interactions between GCs and oocytes modulate gene expression in both cell types, further reinforcing the link between GC transcriptomic signatures and their functional status [2, 8]. As a result, gene expression profiles have become valuable predictors of fertility outcomes in goats [2, 8]. Transcriptomic profiling has emerged as a powerful strategy to address the limitations of conventional GC assessment methods, providing molecular-level insights into the mechanisms of follicular and oocyte development. Among these technologies, single-cell RNA sequencing (scRNA-seq) stands out for its ability to generate high-resolution data on gene activity at the individual cell level. Advances in goat scRNA-seq research have revealed distinct gene expression signatures in GCs that correlate with fertility, confirming the relevance of this technique for reproductive studies [8, 9].

Compared to traditional, non-molecular approaches that provide bulk measurements of hormone levels or protein markers, scRNA-seq offers a much more nuanced view of GC diversity. It distinguishes subtypes of GCs across different follicular stages, traces their developmental trajectories, and reveals dynamic transcriptional changes during follicular maturation. It also uncovers regulatory networks and fertility-associated marker genes that remain undetected with conventional techniques [9–11].

Gene expression profiling enables the identification of differentially expressed genes (DEGs) that may serve as biomarkers of fertility. Recent studies by Xu *et al*. [8] and Ding *et al*. [12] using scRNA-seq have compared DEGs between high-prolificacy (polytocous) and low-prolificacy (monotocous) goat breeds, identifying genes associated with reproductive traits. Despite these findings, directly applying DEGs as biomarkers for fertility assessment presents challenges, especially when trying to develop a quantifiable grading system to evaluate fertility across goat samples. Although DEG expression levels differ between GCs supporting high and low fertility, these differences are not easily translated into a standardized grading framework. A robust evaluation system would need to account for dynamic DEG expression patterns at the single-cell level, necessitating a more advanced strategy to accurately assess GC quality.

In the ovaries of polytocous goats, the presence of both fertility-supporting GCs (FS-GCs) and non-fertility-supporting GCs (NFS-GCs) is expected, with a higher FS-to-NFS ratio promoting the development of multiple follicles and larger litter sizes [2, 8]. Conversely, monotocous goats, which typically produce a single oocyte per cycle, are expected to have a lower proportion of FS-GCs. This difference in GC composition can affect the sensitivity of differential gene expression (DGE) analysis. When FS-GCs and NFS-GCs coexist within the same sample, transcriptomic contrasts between monotocous and polytocous groups become less pronounced, leading to the detection of fewer DEGs due to population heterogeneity.

Although DGE analysis provides important insights, no standardized, gene expression-based metric currently exists for assessing GC quality in goat fertility evaluation. Developing such a tool could significantly improve the precision and efficiency of breeding programs. In response to this need, deep learning offers a promising solution for modeling high-dimensional DEG expression data. Deep learning models such as convolutional neural networks (CNNs) and recurrent neural networks (RNNs) are particularly adept at uncovering complex patterns in gene expression data without requiring manual feature selection [13]. These models also incorporate dimensionality reduction methods, such as autoencoders, which preserve essential biological signals while removing noise, ultimately improving the fidelity of fertility assessments derived from gene expression profiles [14].

Among deep learning architectures, one-dimensional CNNs (1DCNNs) are particularly suited for analyzing sequential data such as DEG profiles. These models can automatically learn spatial features and eliminate irrelevant signals, enabling them to distinguish between FS-GCs and NFS-GCs effectively [15]. In addition, gated recurrent units (GRUs) – a variant of RNNs – are well-equipped to model temporal dependencies in gene expression dynamics. GRUs track cellular state transitions and preserve the sequential integrity of expression profiles, offering deeper insight into GC quality [14]. Integrating 1DCNNs with GRUs in a hybrid architecture enables the simultaneous capture of spatial patterns and temporal dynamics, thereby enhancing the accuracy of GC classification.

Wolf *et al*. [16] have demonstrated that hybrid 1DCNN-GRU models outperform traditional machine-learning approaches in classifying gene expression data, making them particularly well-suited for fertility prediction tasks based on DEG signatures. Furthermore, the hybrid model supports a quantitative framework by predicting the proportion of FS-GCs within a population, providing a scalable metric for assessing cellular quality in reproductive biology.

Despite significant advances in single-cell transcriptomic profiling and its application in reproductive biology, a major gap remains in translating DGE data into practical, quantifiable tools for fertility assessment in goats. The current studies have identified numerous fertility-associated DEGs by comparing GCs from monotocous and polytocous goats; however, these findings have largely remained descriptive or correlation-based. No standardized metric or computational model currently exists to classify GC subpopulations (i.e., FS vs. NFS) at the single-cell level using gene expression data. Furthermore, conventional DEG-based comparisons do not account for intra-sample cellular heterogeneity, leading to reduced sensitivity and potentially misleading conclusions in differential analyses. While deep learning models have shown promise in other areas of transcriptomic classification, their application to reproductive cell-type classification in livestock, especially integrating temporal and spatial patterns in gene expression, has not been explored. There is, therefore, a critical unmet need for a robust, biologically informed, and scalable framework that can classify GCs based on their fertility potential using high-resolution transcriptomic data.

This study aimed to develop and validate a hybrid deep learning model that integrates 1DCNNs and GRUs to classify caprine GCs into FS and NFS categories using scRNA-seq data. By leveraging DEGs identified between monotocous and polytocous goat samples, the model was trained to recognize complex gene expression patterns indicative of cellular fertility function. This work not only provides a proof-of-concept for deep learning-based reproductive cell classification in goats but also introduces a potential quantitative metric for evaluating GC quality. Ultimately, the proposed framework may support future integration into breeding programs as a transcriptomic-based fertility grading tool and could be extended to other livestock species for broader applications in reproductive precision management.

## MATERIALS AND METHODS

### Ethical approval

No ethical approval was required as the study exclusively used previously published scRNA-seq datasets (SRR9945436 and SRR9945437) obtained from the National Center for Biotechnology Information Sequence Read Archive database (https://www.ncbi.nlm.nih.gov/sra).

### Study period and location

This study was conducted between April 2024 and September 2024. All computational analyses were performed using the high-performance server infrastructure provided by the Office of Academic Resources and Information Technology, Rajamangala University of Technology, Tawan-OK, Thailand.

### Sample datasets

This study analyzed scRNA-seq datasets of GCs derived from monotocous (SRR9945436) and polytocous (SRR9945437) goats. The monotocous goat has a consistent history of producing a single offspring per parity. On the contrary, the polytocous goat was 3 years old that had a documented history of at least triplet births per parity over three generations of breeding records. Both goats underwent estrous synchronization through controlled internal drug release for 17 days.

### Experimental design

**Figure 1:**
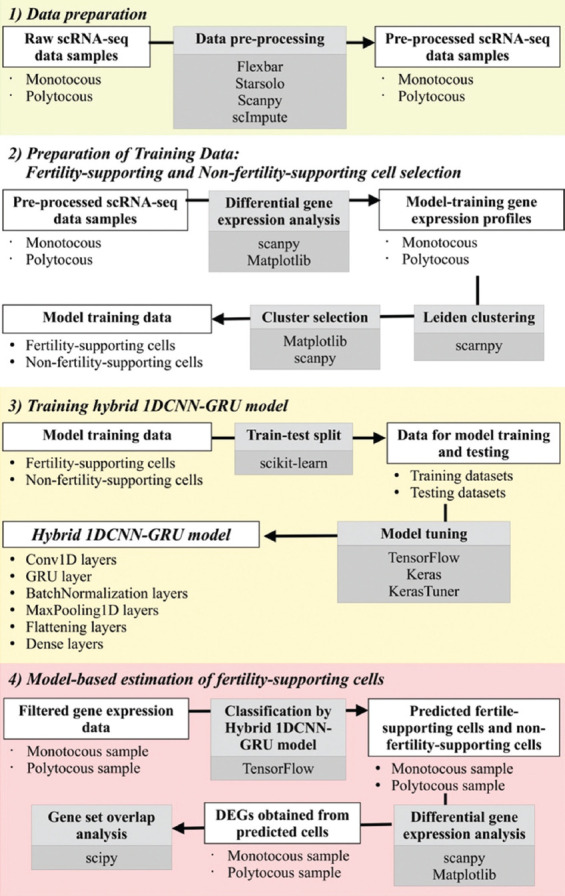
Overview of the analytical workflow. The workflow consisted of four main components: (1) Data preparation, (2) preparation of training data: fertility-supporting and non-fertility-supporting cell selection, (3) training hybrid 1DCNN-GRU model, and (4) model-based estimation of fertility-supporting cells. Each component covered specific analytical processes involving distinct data types as inputs and outputs. The white rectangles indicate the data types used as inputs or generated as outputs, and the gray rectangles represent the analytical processes, including the names of the programs or packages used. 1DCNN-GRU=One-dimensional convolutional neural networks-gated recurrent unit.

Figure 1 illustrates the workflow of our study, comprising four main components:

1. Data preparation
2. Preparation of training data: FS-GCs and NFS-GCs selection
3. Training a hybrid 1DCNN-GRU model
4. Model-based estimation of FS-GCs.

In the “(1) Data Preparation” section, we pre-processed all scRNA-seq datasets, transforming them into gene expression profiles of monotocous and polytocous goat samples. The “(2) Preparation of training data: Fertility-supporting and Non-fertility-supporting cell selection” section utilized the prepared gene expression profiles to identify FS-candidate cell populations and NFS-candidate cell populations, and subsequently, all expression profiles were used to train the hybrid 1DCNN-GRU model in the “(3) Training hybrid 1DCNN-GRU model” section. The trained model was subsequently applied back to monotocous and polytocous samples’ expression profiles in the “(4) Model-based estimation of fertility-supporting cell percentages” section, enabling the quantification of FS-GCs and NFS-GCs in each sample.

#### Data preparation

The preprocessing of raw scRNA-seq datasets was conducted following established protocols. Briefly, low-quality sequences and adapter contamination were removed using the “Flexbar” program (version 3.5.0) [17]. The filtered sequences were then aligned to the Agricultural Research Service 1 reference genome (RefSeq accession: GCF_001704415.1) using “STARsolo” software (version 2.7.10a) [18]. Downstream processing was performed using the “scanpy” toolkit (version 1.9.6) [19], with a subsequent imputation process using the “scImpute” library (version 0.0.9) [20]. Only cells with a minimum total count of 10,000, at least 1,500 detected genes, and a mitochondrial read fraction below 25% were retained.

#### Preparation of training data: FS and NFS cell selection

To identify FS-GCs and NFS-GCs candidates in the preprocessed scRNA-seq samples, we applied Leiden clustering as a semi-supervised approach using DEG expression profiles through the “scanpy” toolkit (version 1.9.6) [19]. First, transcript-per-million normalization was performed, followed by DGE analysis between monotocous and polytocous samples using a modified t-test with thresholds of log2 fold change ≥1.5 and FDR ≤0.01. We refer to these DEGs as “Model-Training DEGs.”

The identified model-training DEGs were then used to construct a concatenated expression matrix combining GCs from both sample types (model-training gene expression profiles in Figure 1), which underwent Leiden clustering [21] with a resolution parameter equal to 0.5. We selected a resolution parameter of 0.5 because this value produced clusters with the highest relative enrichment of either polytocous or monotocous GCs, thereby facilitating a clear distinction between FS-GCs and NFS-GCs. The clustering results were visualized using a two-dimensional Uniform Manifold Approximation and Projection plot.

We considered only clusters with at least 75 valid cells to avoid overinterpreting small, potentially spurious clusters. Clusters comprising ≥80% of the cells in the polytocous sample were classified as FS-cell clusters. In comparison, clusters with ≥80% of the cells from the monotocous sample were designated as NFS- cell clusters. The model-training DEG expression profiles of FS-GCs and NFS-GCs identified by Leiden clustering were subsequently used to train a classification model.

#### Training the hybrid 1DCNN-GRU model

#### Overview of 1DCNN and GRU models

The combination of 1DCNN and GRU networks exploits the strengths of both convolutional and recurrent architectures to effectively analyze sequential data [22, 23]. In this study, we employed this hybrid approach to classify FS-GCs and NFS-GCs based on their expression profiles of Model-Training DEGs.

A 1DCNN processes sequential data by scanning input sequences to identify spatial patterns and local dependencies within numerical sequences, such as expression profiles of Model-Training DEGs [24]. This enables the detection of high-level features essential for distinguishing between FS-GCs and NFS-GCs.

In contrast, the GRU component captures temporal dependencies, making it particularly effective for modeling sequential relationships in gene expression data. Through its gating mechanisms – update and reset gates – the GRU regulates the information flow, ensuring the retention of biologically relevant signals while preventing gradient vanishing during training.

Integrating the 1DCNN and GRU layers enhances model performance by leveraging their complementary functions. The 1DCNN layer extracts localized features, thereby reducing input complexity before passing the data to the GRU, which then captures long-range dependencies. This preprocessing minimizes gradient decay and stabilizes training, thereby improving the efficiency and classification accuracy, particularly for FS-GC classification in this study.

#### Hybrid 1DCNN-GRU architecture

The hybrid 1DCNN-GRU model was optimized to classify GCs as either FS-GCs or NFS-GCs. The architecture of our hybrid model consisted of sequential 1DCNN layers for feature extraction (Table 1), followed by GRU layers to model their temporal relationships (Table 2). The GRU output was reshaped into a one-dimensional vector using a flattened layer before being processed by fully connected (Dense) layers, which synthesized the extracted features for classification. The final cell classification was determined using an activation function that distinguished FS-GCs from NFS-GCs.

**Table 1:** Components presented in 1DCNN architecture.

Components	Summarized details	Equations
Convolutional component	The component processed the sequential expression values of differentially expressed genes using convolutional filters to identify meaningful patterns or motifs in the data. Each filter was defined by its width (kernel size) and scanned across the input sequence to analyze a specified number of data points. These filters adaptively learned weights and biases to emphasize critical features while suppressing noise or irrelevant information. By aggregating the results from all filters, the model generated feature maps representing the presence and intensity of specific patterns across the sequence. This process employed Conv1D layers, where the filters and kernel sizes were tuned to effectively extract features by identifying recurring or localized patterns within the gene expression data.	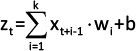 Where: x_t+i-1_ is the input at position t+i-1. w_i_ is the weight of the i^th^ filter. b is the bias term. k is the kernel size.
Normalization component	This component incorporates BatchNormalization layers following the Conv1D layers to standardize data across training batches. Batch normalization was instrumental in stabilizing and accelerating the training process by reducing internal covariate shifts, ensuring that the input to each layer maintained consistent mean and variance. This standardization enhanced the optimization efficiency, contributed to smoother gradient updates, and reduced the risk of overfitting, thereby improving the overall model performance.	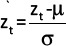 Where: µ is the Mean of the mini-batch. σ is the standard deviation of z_t_.
Pooling component	The pooling component is responsible for dimensionality reduction and focuses on the most critical features in the data. The proposed method employs MaxPooling1D layers, which reduces the dimensionality by selecting the maximum value within the defined windows (pool size) of the input data. This operation effectively downsampled the data, preserving the most significant features while discarding less relevant information. By summarizing each window, MaxPooling1D facilitated computational efficiency and emphasized dominant patterns, which contributed to the model’s ability to learn representative features effectively.	a_p_ = max at, a_t+1_,., a_t+p_ Where: a_t_ is the output after applying the activation function. a_p_ is the result of max pooling operation over a window of size p.
Dropout component	The dropout component mitigated overfitting by randomly deactivating a subset of data units during training. This process introduces stochasticity into the network, preventing reliance on specific neurons and enhancing generalization to unseen data. The modified output with dropout can be expressed as the described equation.	a^‘^_p_=a_p_.m_t_ Where: a_p_ is the result of max pooling operation. m_t_ is a mask vector with elements set to 0 (drop) or 1 (keep) based on the dropout rate. a^‘^_p_ is the resulting activations after applying dropout to a_p_.

1DCNN-GRU=One-dimensional convolutional neural networks-gated recurrent unit

**Table 2:** Components presented in GRU architecture.

Components	Summarized details	Equations
GRU component	The GRU layer processed the sequential features extracted by the one-dimensional convolutional neural network to model the temporal or sequential evolution of these patterns. This approach enabled the network to capture the relationships essential for distinguishing between different cell types and conditions. The GRU architecture consisted of three key components: the Update Gate, Reset Gate, and Candidate Hidden State, which collaboratively managed the information flow: 1. Update gate: This mechanism determines the extent to which past expression values of DEGs influence the current hidden state. The proposed model selectively retained critical information from prior states, thereby ensuring continuity when modeling sequential dependencies. 2. Reset gate: This gate allows the model to focus on new gene expression patterns by controlling the influence of past states. When activated, the GRU was able to disregard outdated dependencies and promote adaptive learning of recent gene activity. 3. Candidate hidden state: this represented the potential updated state of gene activity, integrating current DEG expression patterns with selectively retained information from past states. It is a candidate for updating the final hidden state. 4. Final hidden state: This component encapsulates the cumulative effects of gene interactions and regulatory dependencies up to the current time step, providing a dynamic representation of temporal relationships in gene expression data. The GRU design allows it to efficiently model sequential dependencies and adapt to dynamic patterns in gene expression, making it highly effective for tasks such as distinguishing between cell types or identifying condition-specific regulatory mechanisms.	Update gate z_t_ = σ (W_z_ * Norm (h_ (t-1)_) + U_z_ * Norm (x_t_) + b_z_) Reset gate r_t_ = σ (W_r_ * Norm (h_ (t-1)_) + U_r_ * Norm (x_t_) + b_r_) Candidate hidden state 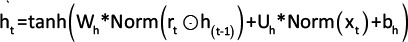 Final hidden state 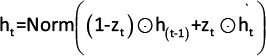 Where: x_t_ The input feature vector at time t, derived from the 1D-CNN layer, represents normalized differential gene expression values. h_t-1_ The hidden state from the previous time step t-1, encoding past information about sequential dependencies. h_t_ The updated hidden state at the current time step t, combining information from h_t-1_, x_t_, and gates. h_t_ The candidate hidden state at time t, represents a potential update for h_t_ based on reset-modulated memory and input. z_t_ The update gate value, controlling the weighting between h_t-1_ and h_t_ in forming h_t_. r_t_ The reset gate value, determining the degree of influence of h_t-1_ on h_t_ during candidate computation. W_z_, W_r_, W_h_ Weight matrices applied to h_t-1_ in the computation of z_t_, r_t_, and h_t_, respectively. U_z_, U_r_, U_h_ Weight matrices applied to x_t_ in the computation of z_t_, r_t_, and h_t_, respectively. b_z_, b_r_, b_h_ Bias terms added to the linear transformations for z_t_, r_t_, and h_t_. σ The sigmoid activation function, 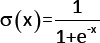 , used for z_t_ and r_t_ calculations. tanh The hyperbolic tangent activation function, tanh (x) = e^x^-e−^x^ e^x^+e^−x^, used for calculation. ⊙ The element-wise (Hadamard) multiplication operator used to apply gate outputs to the hidden state. Norm(.) A normalization function is applied to stabilize inputs or outputs.
Normalization component	This component includes LayerNormalization layers to enhance model stability and performance. By normalizing the input across features, LayerNormalization ensured that the scale of the data remained consistent, which improved the training dynamics and reduced the sensitivity to variations in the input distribution. This standardization step stabilized the data before passing them to subsequent layers, thereby facilitating more effective learning and convergence during model training.	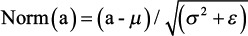 Where: µ Mean of a. σ^2^ Variance of aa. εA small constant to prevent division by 0.

GRU=Gated recurrent unit, DEG=Differentially expressed genes

#### Model training and evaluation

The hybrid 1DCNN-GRU model was trained and evaluated using TensorFlow (version 2.15.0), Keras (version 2.15.0), and KerasTuner (version 1.4.6) [25], with input datasets consisting of FS-GCs and NFS-GCs identified through Leiden clustering [26–28].

To ensure robust performance assessment, the dataset was partitioned into training and testing subsets, with 20% allocated for testing using the train_test_split function from the scikit-learn library (version 1.3.2) (https://scikit-learn.org/stable/index.html). Hyperparameter tuning was conducted using KerasTuner to optimize the model’s performance.

The hyperparameters were tuned by gradually exploring them within predefined ranges. We tuned the number of filters in the initial 1D convolutional layer from 16 to 128 in increments of 4. The number of units in the GRU layer was varied from 16 to 128 in step 4. We tested the fully connected dense layer with values ranging from 16 to 256 in step 4. We tuned the dropout rate as a hyperparameter by testing values between 0.1 and 0.5 in increments of 0.1 to optimize model regularization and prevent overfitting.

We searched the learning rate of the Adam optimizer on a logarithmic scale ranging from 10^−4^ to 10^−2^ to identify the optimal training dynamic. We trained the model using 80% of the data for training and 20% for validation, with stratified sampling to maintain class balance across the splits. We fixed a random seed (42) to ensure reproducibility. We ran the training process for 10 epochs with a batch size of 32, allowing the model to iteratively learn and evaluate its performance on a held-out validation set during each epoch.

During the training process, the model was validated on 30% of the training data to monitor performance. To alleviate overfitting, early stopping regularization was applied, and the model was compiled using the categorical cross-entropy loss function. The Adam optimizer with a learning rate of 0.001 was used for parameter optimization.

The model performance was assessed using accuracy, precision, recall, and F1-score metrics. Predictions for each test sample were generated based on the computed probabilities belonging to either the FS-GCs or NFS-GCs categories. The final performance metrics were calculated using the following equations:

Accuracy = (TP + TN)/Total samples

Precision = TP/(TP + FP)

Recall = TP/(TP + FN)

F1 score = 2 × (Precision × Recall)/(Precision + Recall)

Where:

- True positive (TP): Number of FS-GCs correctly classified
- True negative (TN): Number of NFS-GCs correctly not classified
- False positive (FP): Number of NFS-GCs incorrectly classified as FS-GCs
- False negative (FN): Number of FS-GCs incorrectly classified as NFS-GCs.

#### Model-based estimation of FS cell percentages

The optimized hybrid 1DCNN-GRU model was used to predict class probabilities for each GC in monotocous and polytocous goats. Each cell was classified into the FS or NFS category based on the highest predicted probability using a classification threshold of 0.95 to prioritize high-confidence predictions. We considered GCs falling below the threshold as ambiguous and thus excluded from class-specific interpretation.

DGE analysis was subsequently performed between the predicted FS-GCs and NFS-GCs across monotocous and polytocous samples using the modified t-test method from the scanpy toolkit (FDR ≤0.01 and log2 fold change ≥1.5).

After identifying DEGs in each sample, we used the matplotlib_venn library to generate a Venn diagram to visualize the overlap of DEGs identified in each sample with those used for model training. The observed overlap of DEGs identified in the polytocous and monotocous regions and the original model-training DEGs could be attributed to the biological consistency in gene expression profiles characterizing FS-GCs and NFS-GCs. This overlap (hypergeometric test, p ≤ 0.05) supported the model’s generalizability beyond the training data by indicating biological consistency in the gene expression profiles of FS-GCs and NFS-GCs across samples.

## RESULTS

### DGE in monotocous and polytocous goats

The scRNA-seq data of GCs from monotocous and polytocous goats were successfully pre-processed for downstream analysis. Following data preparation, 1,588 GCs were obtained from the monotocous goat sample and 1,536 from the polytocous goat sample. DGE analysis identified 44 genes (Supplementary Table 1) with statistically significant differences between the two groups (p ≤ 0.01, log2 fold-change ≥1.5). These genes were designated as “Model-Training DEGs” and were used in subsequent clustering and model-training steps.

### Identification of FS and NFS cells through Leiden clustering

**Figure 2:**
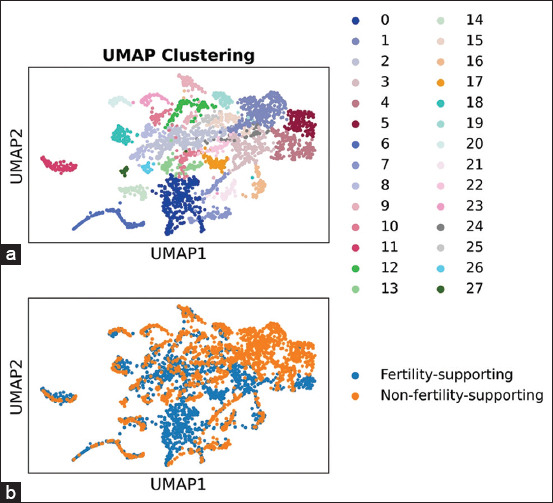
Uniform manifold approximation and projection visualization of granulosa cell clusters and sample types. (a) Granulosa cell clusters identified by Leiden clustering are displayed, each represented by a unique color. (b) The distribution of sample types categorized granulosa cells into fertility-supporting and non-fertility-supporting groups.

Leiden clustering based on the expression profiles of the 44 model-training DEGs revealed 28 distinct GC clusters (Figure 2 and Table 3), each containing varying proportions of cells from the monotocous and polytocous samples. Clusters 0 and 17, each comprising more than 75 cells and with over 80% of their cells derived from the polytocous sample, were designated as FS cell clusters. Conversely, clusters 1 and 5, each with more than 75 cells and >80% of their composition from the monotocous sample, were categorized as NFS clusters.

**Table 3:** Leiden clusters and corresponding polytocous and monotocous granulosa cell counts.

Leiden cluster number	Total number of cells	Polytocous granulosa cells		Monotocous granulosa cells	
	
		Cell number	Percentage	Cell number	Percentage
0	336	315	93.75	21	6.25
1	332	6	1.81	326	98.19
2	298	206	69.13	92	30.87
3	199	144	72.36	55	27.64
4	172	78	45.35	94	54.65
5	152	5	3.29	147	96.71
6	121	71	58.68	50	41.32
7	114	34	29.82	80	70.18
8	112	73	65.18	39	34.82
9	102	38	37.25	64	62.75
10	101	62	61.39	39	38.61
11	97	70	72.16	27	27.84
12	92	35	38.04	57	61.96
13	85	23	27.06	62	72.94
14	83	47	56.63	36	43.37
15	83	19	22.89	64	77.11
16	79	53	67.09	26	32.91
17	76	76	100.00	0	0.00
18	76	49	64.47	27	35.53
19	65	18	27.69	47	72.31
20	56	32	57.14	24	42.86
21	53	23	43.40	30	56.60
22	52	7	13.46	45	86.54
23	51	11	21.57	40	78.43
24	44	12	27.27	32	72.73
25	42	5	11.90	37	88.10
26	31	15	48.39	16	51.61
27	20	9	45.00	11	55.00

**Figure 3:**
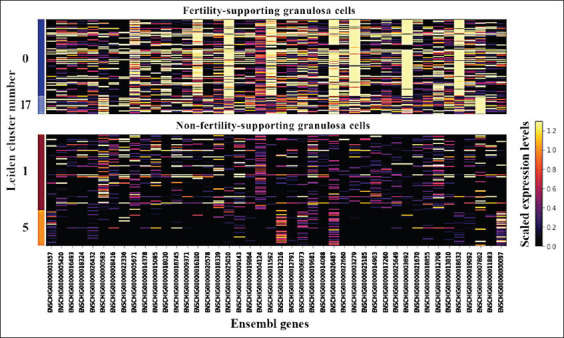
Expression patterns of model-training DEGs in FS-GCs and NFS-GCs clusters. Each row represents a Leiden cluster, with clusters 0 and 17 corresponding to FS-GCs and clusters 1 and 5 corresponding to NFS-GCs. Columns represent DEGs in Ensembl IDs and are color-coded according to their expression levels (scaled from 0 to 1.2 in the color bar). Yellow indicates higher expression levels, whereas dark purple indicates lower expression levels. Noticeable differences in expression patterns were presented between FS-GCs and NFS-GCs clusters, as well as subtle variations within each cluster. FS=Fertility-supporting, NFS=Non-fertility-supporting, DEGs=Differentially expressed genes, GCs=Granulosa cells.

Notable expression differences of model-training DEGs were observed between FS (clusters 0 and 17) and NFS clusters (clusters 1 and 5), as shown in Figure 3. Some variability in expression patterns was also seen within clusters. The scaled expression data from these FS-GCs and NFS-GCs were then used to train a classification model in the next step.

### Training and optimization of the hybrid 1DCNN-GRU model

**Figure 4:**
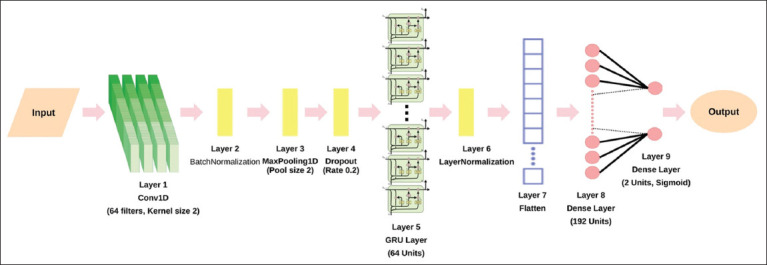
Architecture of the proposed 1DCNN-GRU model for granulosa cell classification. The proposed model began with an input layer, an orange parallelogram corresponding to the input data – DEG profiles from FS-GCs and NFS-GCs. Layer 1 consisted of a 1D Convolutional layer (Conv1D) with 64 filters and kernel size 2, which is visualized as stacked green rectangles. This was followed by BatchNormalization in Layer 2 as a yellow rectangle. Layer 3 applied MaxPooling1D with a pool size of 2, effectively reducing the spatial dimensions of the feature map. Layer 4 includes a dropout layer with a dropout rate of 0.2 to mitigate overfitting. The GRU layer, visualized as stacked GRU cell diagrams, constituted Layer 5 and consisted of 64 units for sequence processing, followed by LayerNormalization in Layer 6 (yellow rectangle) to normalize the outputs of the GRU layer. In Layer 7, the outputs are flattened into a single-dimensional vector for the subsequent fully-connected layer. Layer 8 is a fully connected Dense layer with 192 units. The model concluded with Layer 9, an output-dense layer with 2 units and a Sigmoid activation function, which predicted the probabilities for the two classes: FS-GCs or NFS-GCs. The final model output was an oval with pink arrows indicating the flow through the network. 1DCNN-GRU=One-dimensional convolutional neural networks-gated recurrent unit, FS=Fertility-supporting, NFS=Non-fertility-supporting, DEGs=Differentially expressed genes, GCs=Granulosa cells, GRU=Gated recurrent unit.

Using the DEG expression profiles from FS-GCs and NFS-GCs, a hybrid 1DCNN-GRU model was developed and optimized using Keras Tuner. The final model achieved high classification performance with an accuracy of 98.89%, a precision of 100%, a recall of 97.83%, and an F1 score of 98.84%. The final model architecture is presented in Figure 4, and detailed specifications are summarized in Table 4. In addition, a saliency-based analysis of individual gene contributions to model predictions is provided in Supplementary Table 1.

**Table 4:** Summary of the hybrid 1DCNN-GRU model architecture.

Component	Architecture
1DCNN	Layer 1 - Conv1D (Filters: 64 filters, Kernel size 2): Features extracted from the input data using 64 filters of size 2 with ReLU activation function. Layer 2 - BatchNormalization: Normalized the Layer inputs to improve training stability. Layer 3 - MaxPooling1D (Pool size 2): The dimensionality of the data is reduced by taking the maximum value from every window of size 2. Layer 4 - Dropout (Rate: 0.2): Randomly doped 20% of the activations during training to prevent overfitting.
The GRU	Layer 5 - GRU (Units: 64, Dropout: 0.2, Return Sequences: False): The GRU Layer captured sequential dependencies and temporal patterns in the data processed by the preceding 1DCNN layers. Layer 6 - LayerNormalization: This Layer ensures that the standardized data across features improves stability and convergence during training. Layer 7 - Flatten: Reshapes the data to a one-dimensional vector. Layer 8 - Dense (Units: 192 units): The first fully-connected Layer with 192 neurons and ReLU activation. Layer 9 - Dropout (Rate: 0.2): Randomly doped 20% of the activations during training to prevent overfitting. Layer 11 - Dense (Units: 2): Output Layer with 2 units and sigmoid activation for predicting probabilities of belonging to 2 different classes – fertility- and non-fertility-supporting classes.
Model compilation	Loss: Categorical cross-entropy. Optimizer: Adam optimizer with a learning rate of 0.001. Metrics (to monitor classification performance): Accuracy, Precision, Recall, and F1 score.

1DCNN=One-dimensional convolutional neural networks, GRU=Gated recurrent unit, ReLU=Rectified linear unit

### Model-based estimation of FS-GCs in monotocous and polytocous samples

**Figure 5:**
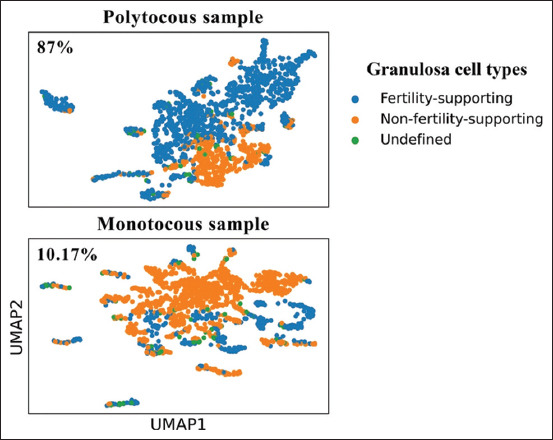
UMAP visualization of granulosa cell classification in polytocous and monotocous samples. UMAP projections of granulosa cells from polytocous (top panel) and monotocous (bottom panel) samples, classified into three categories: fertility-supporting cells (blue), non-fertility-supporting cells (orange), and undefined cells (green). The classification highlighted distinct clustering patterns based on the fertility-supporting capacity of the cells, reflecting biological differences between the two reproductive types. UMAP=Uniform manifold approximation and projection.

The trained 1DCNN-GRU model was applied to classify individual GCs from the monotocous and polytocous samples. Using a confidence threshold of 0.95, the model identified 360 FS-GCs in the polytocous sample and 160 FS-GCs in the monotocous sample, corresponding to 87% and 10.17% of the total cells, respectively (Figure 5).

Further DEG analysis between predicted FS-GCs and NFS-GCs within each sample revealed gene sets that overlapped substantially with the original model-training DEGs. We define “Polytocous DEGs” as DEGs distinguishing FS-GCs within the polytocous sample (194 genes, Supplementary Table 2) and “Monotocous DEGs” as those differentiating FS-GCs within the mono-tocous sample (53 genes, Supplementary Table 3).

**Figure 6:**
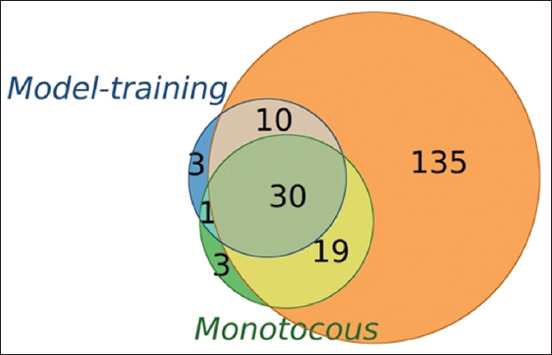
Venn diagram illustrates the overlap between model-training DEGs, Polytocous DEGs, and Monotocous DEGs. The Venn diagram depicts the distribution of DEGs across model-training DEGs (the circle with the blue outline), Polytocous DEGs (the circle with orange outline), and Monotocous DEGs (the circle with the green outline). Notably, most model-training DEGs overlapped with both Polytocous and Monotocous DEGs. In contrast, 135 DEGs were exclusively identified as Polytocous DEGs, highlighting the distinct characteristics of these DEG categories. DEGs=Differentially expressed genes.

As shown in the Venn diagram (Figure 6), 30 DEGs were common among the model-training, polytocous, and monotocous DEG sets. Notably, the polytocous DEG set encompassed nearly all genes found in the other two groups, indicating a broader and more distinct transcriptional profile for FS-GCs in polytocous goats.

## DISCUSSION

### Integrating deep learning with DEG analysis for fertility assessment

DGE analysis of GC scRNA-seq profiles has become a powerful method for evaluating fertility in goats. It enables the identification of DEGs that serve as potential biomarkers, offering insight into ovarian processes crucial for oocyte maturation [8, 12]. In this study, we proposed a novel method that integrates DEG expression data with a hybrid deep learning model – specifically, a 1DCNN-GRU architecture – to assess GC quality. The model was trained using DEGs identified between monotocous and polytocous samples, with FS-GCs and NFS-GCs defined through Leiden clustering. The model demonstrated high classification accuracy and successfully identified a higher proportion of FS-GCs in the polytocous sample, showing its potential as a transcriptomic-based GC grading tool, even for samples with comparable fertility.

### Biological relevance and limitations of the DEG set

The analysis confirmed distinct DEGs between monotocous and polytocous samples, suggesting underlying GC subtype differences. While this finding is promising, the study’s small dataset – comprising only one monotocous and one polytocous sample – limits its generalizability. To strengthen model reliability, future studies should expand sample sizes and include animals with diverse reproductive phenotypes and genetic backgrounds.

Previous studies by Sharma and Sharma [2], Xu *et al*. [8], and Xie *et al*. [11] examining DEGs between GCs from monotocous and polytocous goats have reported key genes associated with litter size and fertility traits. Despite variations in study designs, our findings corroborated several critical DEGs reported in earlier scRNA-seq work, including TGFB2, WNT5B, EDN1, and DES [8]. Functional enrichment analysis of these DEGs indicated significant involvement in translation, cell cycle regulation, and angiogenesis – key processes for GC differentiation and follicular support. In addition, enriched cellular components, such as ribosomal structures and the endoplasmic reticulum, suggested high biosynthetic activity in GCs (Supplementary Figure 1). These findings supported the use of this DEG set for modeling, justifying their label as “Model-Training DEGs.”

### Leiden clustering captures biologically meaningful GC subtypes

Leiden clustering of scRNA-seq data filtered by Model-Training DEGs enabled the identification of biologically distinct GC subtypes. Consistent with emerging single-cell literature, our results support the classification of GCs into FS-GCs and NFS-GCs [8], with a higher FS-GC ratio expected in polytocous samples. Our choice of an 80% threshold to define cluster identity, while somewhat arbitrary, is consistent with best practices in exploratory single-cell studies. Future research could refine this cutoff using larger datasets.

To minimize overfitting and artifacts from scImpute and Leiden clustering, we applied a stricter minimum cell threshold of 75 cells per cluster. This helped reduce the influence of small or potentially spurious clusters. Although intra-cluster variability in DEG expression was observed (Figure 3), the hybrid model was still able to extract discriminative features, indicating the robustness of the training strategy.

### Advantages of the 1DCNN-GRU hybrid model

Our results demonstrate that the hybrid 1DCNN-GRU model offers significant advantages over traditional machine learning methods and standalone deep learning models, such as CNN, GRU, and long short-term memory. The hybrid model’s ability to handle sequential gene expression data without manual feature selection positions it ahead of methods, such as Support Vector Machine and Random Forest [29]. The GRU layer captures temporal dependencies, whereas the CNN layer extracts local features and mitigates noise, improving generalization and training efficiency [29, 30]. This complementary integration enhanced accuracy and model convergence across most evaluations.

### Mechanism of feature extraction and interpretation

The model’s design leverages the strengths of both architectures: 1DCNNs extract sharp expression trends or gene activation patterns [31, 32], whereas GRUs capture spatial and sequential dependencies across DEGs [32]. Dimensionality reduction through pooling preserved relevant features without distortion, allowing the GRU layers to track gene expression dynamics critical for cell classification. Consistent with this rationale, the model estimated FS-GC proportions of 87% in the polytocous and 10.17% in the monotocous sample (Figure 5), supporting its application as a molecular fertility assessment tool.

### Biological implications of within-sample DEG analysis

GC composition heterogeneity reduced DEG detection sensitivity in inter-group comparisons, as the presence of both FS-GCs and NFS-GCs diluted transcriptomic contrasts. However, within-sample DEG analysis – particularly in polytocous samples – revealed larger and more distinct DEG sets, indicating clearer expression differences between FS-GCs and NFS-GCs. Notably, the 194 “Polytocous DEGs” (Supplementary Table 2) captured nearly all genes included in the 44 model-training DEGs and the 53 “monotocous DEGs” (Supplementary Table 3), as shown in Figure 6.

### Conserved fertility-associated transcriptional signatures

Thirty genes were shared among all three DEG groups (Model-Training, Polytocous, and Monotocous DEGs), suggesting the presence of a conserved core transcriptional program underlying GC fertility. These genes likely play essential roles in GC function and oocyte support and may be broadly applicable as fertility biomarkers. The broader transcriptomic divergence observed in polytocous samples may indicate higher GC subtype complexity, potentially linked to enhanced reproductive potential [8].

### Ovarian heterogeneity and its functional relevance

Our findings support the concept that ovarian cellular heterogeneity, including subtype-specific GC profiles, may significantly influence reproductive traits. This heterogeneity may play a key role in follicular development and oocyte maturation. Future studies should use advanced lineage tracing and functional assays to validate the roles of individual subtypes. Identifying and targeting subtype-specific gene markers may open avenues for precision reproductive diagnostics and interventions in goat breeding.

### Model utility and future applications

This study highlights the utility of deep learning in translating scRNA-seq data into biologically meaningful fertility assessments. Although biological validation was beyond the scope of this study, the model lays the groundwork for future validation and translational research. The ability to compute a fertility score based on FS-GC proportions offers a promising tool for reproductive management. With further validation, this model could complement traditional fertility metrics in selecting high-fertility donors, optimizing mating plans, or screening reproductive health in goats and other livestock species.

## CONCLUSION

This study presents a novel deep learning-based framework for evaluating GC fertility potential in goats by integrating scRNA-seq data with a hybrid 1DCNN-GRU model. By leveraging 44 DEGs (model-training DEGs) identified between monotocous and polytocous goat GCs, the model was trained on biologically validated subpopulations of FS and NFS GCs defined through Leiden clustering. The optimized model achieved a classification accuracy of 98.89%, with a precision of 100%, a recall of 97.83%, and an F1 score of 98.84%, demonstrating exceptional performance in classifying GCs based on fertility-associated transcriptomic profiles.

Importantly, the model predicted a significantly higher proportion of FS-GCs in the polytocous sample (87%) compared to the monotocous sample (10.17%), consistent with known biological expectations. This ability to quantify FS-GC ratios introduces a practical and scalable method for molecular-level fertility assessment, offering potential utility in reproductive management, the early selection of high-fertility individuals, and the screening of donor suitability for embryo transfer programs.

A major strength of this study lies in its methodological innovation, which integrates spatial and temporal pattern recognition through a 1DCNN-GRU hybrid architecture while minimizing manual feature engineering. The approach also incorporated robust clustering strategies and conservative thresholds to reduce overfitting and false classifications, thereby increasing the biological reliability of cell subtype identification.

Nonetheless, the study has limitations. The analysis was based on a limited dataset (one monotocous and one polytocous goat), restricting the generalizability of the findings. Further, the model’s predictions, while biologically plausible, have not yet been validated through functional assays or lineage tracing.

Future research should focus on expanding the dataset across diverse genetic backgrounds, reproductive phenotypes, and developmental stages. Functional validation of FS-GC markers, refinement of clustering thresholds, and incorporation of additional cell types (e.g., theca or stromal cells) could further enhance the model’s predictive power. Integration of such a system into practical fertility screening tools or breeding decision platforms would offer significant benefits to livestock production.

In conclusion, this study provides proof-of-concept for using deep learning to evaluate GC fertility at single-cell resolution. It bridges a critical gap between transcriptomic data and actionable reproductive metrics, paving the way for precision breeding in goats and potentially other livestock species.

## DATA AVAILABILITY

Supplementary tables and figures can be available from the corresponding author. Supplementary Table 1 presents a list of model-training DEGs with Ensembl gene IDs and gene symbols, along with their respective saliency scores indicating their contribution to the classification of FS-GCs and NFS-GCs. The lists of polytocous DEGs and monotocous DEGs, which were also annotated with Ensembl gene IDs and gene symbols, are available in Supplementary Tables 2 and 3, respectively. Supplementary Figure 1 presents the gene ontology term enrichment analysis of the model-training DEGs.

## AUTHORS’ CONTRIBUTIONS

TS and KC: Conceptualized and designed the study, performed the experiments, and drafted the manuscript. TS, DP, and KC: Analyzed the data and revised the manuscript. All authors have read and approved the final manuscript.
